# Individual-, social- and policy- factors associated with smoking cessation among adult male cigarette smokers in Hanoi, Vietnam: a longitudinal study

**DOI:** 10.1186/s12889-023-16781-7

**Published:** 2023-09-28

**Authors:** Thi Ngoc Phuong Nguyen, Jesper Love, Monica Hunsberger, Thi Phuong Thao Tran, Thuy Linh Nguyen, Thi Hai Phan, Ngoc Khue Luong, Van Minh Hoang, Nawi Ng

**Affiliations:** 1 School of Public Health & Community Medicine, Institute of Medicine, Sahlgrenska Academy, University of Gothenburg, 41390 Gothenburg, Sweden; 2 Center for Population Health Sciences, Hanoi University of Public Health, Hanoi, Vietnam; 3 Ministry of Health, Hanoi, Vietnam; 4 Department of Epidemiology and Global Health, Faculty of Medicine, Umea University, Umea, Sweden

**Keywords:** Cessation, Intention to quit, Policy, Social behavior

## Abstract

**Background:**

Nearly one-in-two Vietnamese men smoke cigarettes placing them among the highest tobacco consumers in the world. Despite the need for smoking cessation to curb the burden of tobacco-related diseases in Vietnam, this rate remains at less than 30%. Therefore, this study examines individual-, social- and policy factors associated with smoking cessation among adult male smokers in Vietnam.

**Methods:**

We established a longitudinal International Tobacco Control study of male smokers in Hanoi, Vietnam, in September 2018. This paper analyses 1525 men who participated in baseline and one-year follow-up. We applied a weighted multivariable logistic regression to examine the association between smoking cessation and individual-, social- and policy predictors.

**Results:**

At follow-up, 14.8% of participants had quit smoking for at least 30 consecutive days during the last year. Among the persistent smokers, 56.6% expressed intention to quit smoking. Factors associated with smoking cessation included a lower number of cigarettes smoked per day (aOR = 0.96, 95% CI: 0.94, 0.99) and having several attempts to quit smoking (aOR = 2.16, 95% CI 1.13, 4.12). Intention to quit smoking was associated with multiple quit attempts, a chronic condition diagnosis, more tobacco-related knowledge, greater self-efficacy, and more worries about their future health. The perceived impact of smoke-free policy and health warning labels were positively associated with intention to quit at any stage.

**Conclusions:**

Interventions aimed at increasing smoking cessation should focus on all aspects of individual, social, and policy factors. Persistent smokers are more motivated to quit if they have made multiple quit attempts, more self-efficacy of quitting and worried about their future health, indicating that increasing smokers’ beliefs and knowledge may be important for behavioural change. Health warning labels and tobacco taxation policies should be maintained and promoted as they are perceived to be particularly useful for persistent smokers’ intention to quit.

**Supplementary Information:**

The online version contains supplementary material available at 10.1186/s12889-023-16781-7.

## Introduction

Vietnam is a low-middle-income country where nearly half of the men consume tobacco products [1]. According to the 2015 Global Adult Tobacco Survey (GATS), of the men that consumed tobacco, more than 85.0% did so daily, and mainly in the form of cigarette [1]. Despite efforts to reduce smoking prevalence, the cessation rate remained largely unchanged between 2010 and 2015 [1, 2].

The addictive and harmful nature of nicotine necessitates interventions aimed at reducing smoking prevalence. Of 1.1 billion all-type tobacco smokers globally [3], about two-thirds intended to quit, and more than 40% attempted to quit smoking [4]. It is widely recognized that quitting smoking at any age can lead to overall health improvement and reduce the risk of smoking-related diseases [5]. In fact, smokers can even reduce their risk of tobacco-related premature deaths by up to 90.0% if they quit before the age of 40 [6]. Earlier studies have shown that intention to quit and smoking cessation are associated with older age (over 55 years old) [7, 8], being in an early stage of nicotine addiction [7–9], having the previous quit attempts [7–9], and being affected by tobacco control policies [10].

Nearly 30 years ago, the Vietnamese government recognized the need for tobacco control policies. The initial actions began already in 1986 when cigarettes were banned for youth under 15 years of age. Later, the first comprehensive legal framework for tobacco control in Vietnam in 2000 [2, 11]. This framework established a department called the Vietnam Steering Committee on Smoking and Health (VINACOSH) aimed at controlling and coordinating tobacco activities. Then, in 2003, the World Health Organization (WHO) Convention on Tobacco Control Framework was signed, which was later ratified at the end of 2004, and officially went into effect in March 2005 [2]. The National Assembly of Vietnam approved the MPOWER package and introduced the first national Tobacco Control law in June 2012, which took effect one year later [12].

Despite achievement numerous progressive tobacco control policies in Vietnam during this time (i.e., health warning labels, bans on tobacco advertising and a 15% increase in tobacco taxes), the national tobacco smoking prevalence decreased from 56.1% in 2001 to 45.3% in 2015 [1, 2, 13]. Since the prevalence remains high, efforts are placed on smoking cessation. However, a 2015 national survey found that 42.4% of current smokers had no interest in quitting tobacco; and only half had intention to quit in the future [1].

Further, the smoking cessation rate remained unchanged at 29.0% from 2010 to 2015 according to the GATS surveys [1]. Evidence in cigarette smoking cessation behaviour and the related individual, social and policy factors among Vietnamese men are limited. The existing studies have examined cessation behaviours using the cross-sectional data only, and none have examined the role of social and policy factors on smoking behaviour change [1, 2, 14]. Further, previous studies conducted in other countries found some individual factors associated with smoking cessation in Western or Asian countries only, where the culture and policies are different from Vietnam (including the social norms or the implemented level of tobacco control policies). Also, earlier studies only covered one or two aspects (individual, social, or policy) associated with smoking cessation, while they together influence smokers’ behaviour. Therefore, this study examines the individual-, social- and policy- factors associated with smoking cessation among adult male smokers in Vietnam.

## Methods

### Study design

This study is part of the International Tobacco Control Policy Evaluation Project (the ITC Project), the first international cohort study of tobacco use. The ITC Project is a collaborative effort with international health organizations and policymakers in 31 countries. Using standardized ITC protocol, we recruited a sample of adult male smokers in Hanoi, Vietnam. Baseline survey data was collected at the beginning of September 2018 and follow-up data was collected approximately one year later. Ethical approval for this study was obtained from the Institutional Review Board of Hanoi University of Public Health (No 419, 422/2018/YTCC-HD3 for the baseline study and No 474/2019/YTCC-HD3 for the follow-up study).

### Study sample and sampling method

We recruited the respondents from households using a stratified multistage sampling design. We defined the strata by geographic region and community size in four steps. First, two districts of Hanoi (Cau Giay district—representing urban areas and Quoc Oai district—representing rural zones) were purposively selected since they were in close consultation with the Vietnam Steering Committee on Smoking and Health and expressed commitment to tobacco control activities. Second, in the two districts, each ward/town/commune had a list of all neighbourhood, so-called primary sampling units (PSU). These PSUs were defined based on their population sizes from the 2009 Vietnam Population and Housing Census conducted by the General Statistics Office of Vietnam [15]. After that, we randomly selected 34 PSUs in urban and 30 PSUs in rural districts from the list of PSUs. Then local authorities provided a list of households in each randomly selected PSU. A local health worker contacted each household on their list and produced a list of households with smokers and without smokers. We then used the list of households with smokers in each PSU to create the sampling frame. Third, households with smokers were chosen at random in each PSU based on the population size in the cluster. On average, there were 29 households (range: 13–56) in the selected urban PSU and 33 households (range: 16–68) in the selected rural PSU. Finally, only one smoker from each household who met the inclusion criteria described below was invited to participate. If the household had more than one eligible smoker, we applied the next-birthday method to select one of these smokers [16]. In case the selected participant refused to participate, another eligible smoker in the same household or the next household was invited to participate. The refusal rate in the baseline survey was less than 5.0%. Detailed information about the sampling method and sample size calculation is described in the technical report of ITC Vietnam [17].

This multistage cluster sampling approach resulted in a random sample of adult male smokers from the two districts in Hanoi. We recruited the participants who met all of the following inclusion criteria: (1) male smokers; (2) had smoked more than 100 cigarettes in their lifetime and are currently smoking at least once a week; (3) 18 years of age or older; (4) residents of Hanoi, Vietnam and who do not intend to migrate to other areas in the next three years; (5) free from any mental disease; and (6) could read and understand all provided information and consented to participate in our study. Consistent with the ITC protocol, our target sample size at baseline was 1000 smokers in each sampling area, for a total of 2000 smokers. This sample size was robust enough to assess any statistically significant changes in the smoking behaviours and ensure a sufficient sample in case of attrition in this longitudinal study [18]. This sample size was also applied in other countries implementing the ITC Project and mentioned in its protocol [18].

**Fig. 1:**
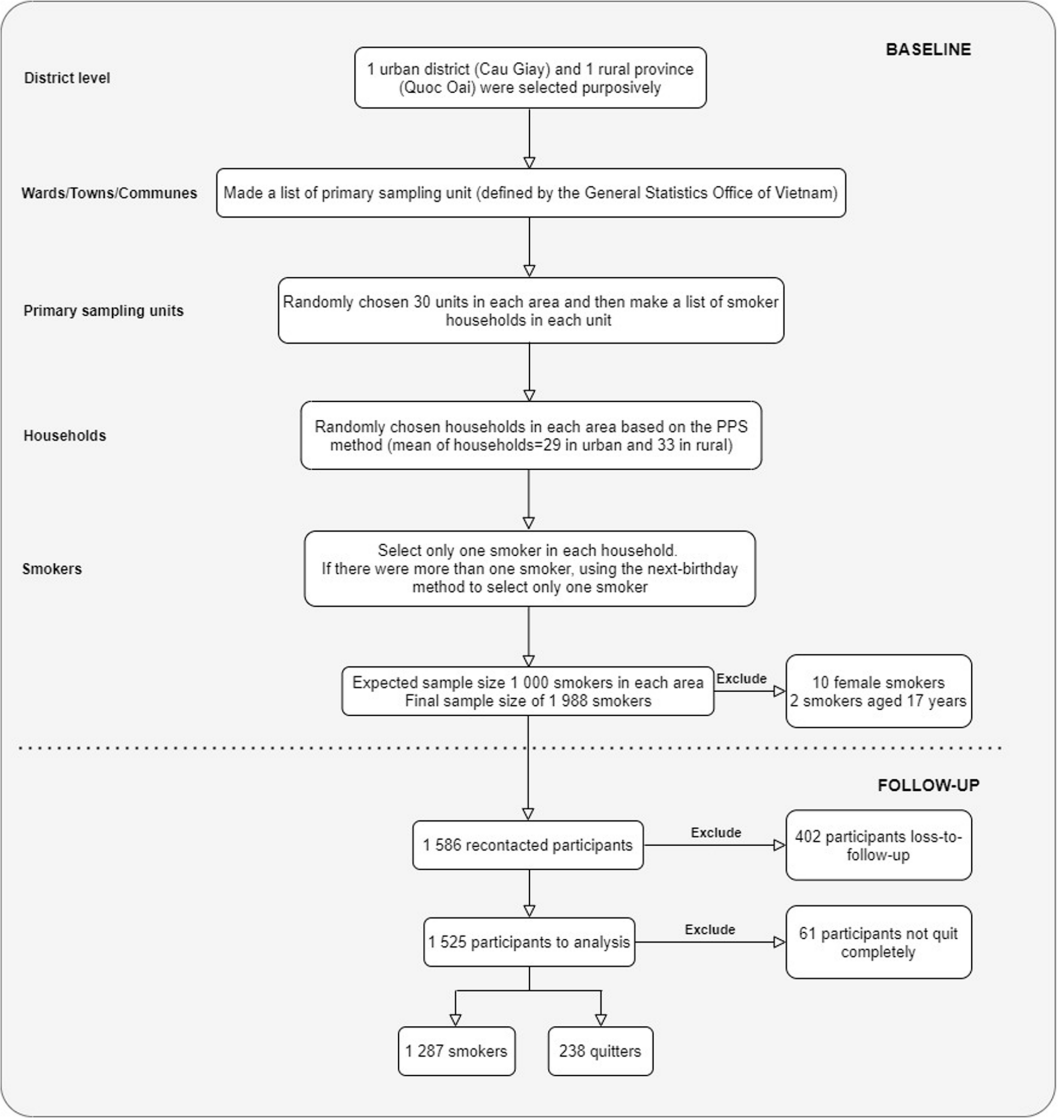
A flow chart of a sample selection procedure

After removing the incomplete responses, we retained 1988 smokers from the baseline survey. At one-year follow-up, 1585 of 1988 participants agreed to participate, resulting in a 79.8% response rate. After excluding 60 people who quit cigarette smoking but changed to other forms of tobacco, we obtained a final analytical sample of 1525 participants. The details of the sample selection procedure are shown in Fig. 1.

### The survey instrument and data collection

The Center for Population Health Sciences, Hanoi University of Public Health, researchers designed the questionnaire based on the ITC protocol and guided by the GATS survey that was conducted in Vietnam in 2010 and 2015 [1, 18]. The study was carried out through in-person interviews conducted by a team of public health students, in collaboration with the district Center for Preventive Medicine. Prior to the interviews, participants were informed about the study and asked for their written consent to participate. During the data collection, participants were made aware of their right to withdraw from the study and were assured that their personal details would be removed from the final dataset for analysis.

### Variables

#### Dependent variables

The primary outcome measure was smoking cessation behaviours. We categorized the participants as either “quitters” or “persistent smokers” based on their smoking behaviours at a one-year follow-up. Quitters were participants who quit tobacco smoking (both cigarette and other tobacco products) for at least 30 consecutive days prior to data collection at a one-year follow-up. We calculated the proportion of smoking cessation as the percentage of quitters out of the total participants in the baseline. We categorized participants as persistent smokers if they continued or did not quit for less than 30 consecutive days before data collection at one-year follow-up.

Besides, we defined smokers with quit attempts if they tried to quit smoking and their intention to quit smoking if they expressed their plan to quit smoking sometime in the future. This information was measured at both baseline and one year follow-up. Guided by the transtheoretical model of health behaviour change, we further categorized their intention to quit as those in the preparation (if they intended to quit within the next month), contemplation (if they intended to quit within the next six months), and pre-contemplation stage (those who planned to quit in the future) [19].

#### Independent variables

Key variables were assessed at baseline for socioeconomic factors (e.g., age, occupation, education level, household income, and residential area), and at follow-up for health behaviour (number of quitter friends/acquaintance, quality of life). Quality of life was measured using a visual analogue scale (VAS) ranging from 0 to 100, where 0 means the worst health and 100 means the best health. We also collected information about their self-reported health status, household socioeconomic status and household assets at both baseline and follow-up, including the household’s classification by the government into poor, near-poor and average households based on their monthly income. For smoking behaviour, we collected the following information at baseline and follow-up (for persistent smokers): the average number of cigarettes smoked per day (CPD), the number of smoker/quitter friends/family members, intention to quit, and tobacco-related knowledge. To measure the level of nicotine dependence, we calculated a six-point heaviness smoking index (HSI) by combining information about the CPD (scored as 0: 1–10 CPD, 1: 11–20 CPD, 2: 21–30 CPD, 3: ≥ 31 CPD) and the time to the first cigarette after waking (scored as 3: less than/equal to 5 min, 2: 6–30 min, 1: 31–60 min, and 0: ≥ 61 min). We categorized smokers into either cigarette smoking only or dual users (smoked more than one tobacco product). Further, tobacco-related knowledge was measured by asking 14 questions related to the participants’ awareness of tobacco-related diseases at both baseline and follow-up. The higher the knowledge score, the more knowledgeable the participants were. We categorized the knowledge score into quartiles, with the first quartile representing the 25% lowest score and the last quartile representing the 25% highest score.

We included four variables to measure the participants’ attitudes and beliefs toward smoking and cessation at the baseline survey and follow-up survey. These variables were self-efficacy to quit smoking, health benefits of quitting, worry about future health, and opinion of smoking. We further asked the participants to assess their perceived impact of tobacco control policies in Vietnam on their cessation behaviour. The policies included smoke-free policies in public environments, cessation support programs, health warning labels on cigarette packaging, anti-smoking advertising, and tobacco taxation. These variables were assessed at the follow-up survey only, except for health warning labels on cigarette packaging and anti-smoking advertising. Detailed information on these attitudes/beliefs and their perceived impacts on policy variables are presented in Supplementary Table 1. The full questionnaire is available on the ITC website of the ITC Vietnam project [17].

### Statistical methods

In this study, the baseline weight was constructed from all four levels of sample selection, including individual, household, PSU, and ward/commune. The longitudinal weight was calculated for all recontacted participants but scaled to adjust the attrition based on the baseline weight. Unless otherwise noted, all descriptive and regression results were weighted using the longitudinal weight. The complete study details, including the weighting procedure, are presented in the ITC Vietnam technical report [17].

We conducted descriptive analyses and presented the smoking cessation prevalence across different population subgroups. These subgroups were grouped as the socioeconomic status, smoking and health behaviours, beliefs/attitudes/opinions of smoking, and perceived impacts of the implemented tobacco control policies. We presented the results as numbers and percentages for categorical variables or mean and standard error (SE) for continuous variables.

The number of households grouped as poor or near-poor based on the government’s nationwide classification in our sample was small; the variable was considered non-discriminatory to differentiate household socioeconomic status. Therefore, we used principal component analysis (PCA) to create a household wealth index based on household assets variables [20]. These variables included housing characteristics (including the house’s roof and wall materials, sources of drinking, and types of latrines) and household durable assets (TV, fridge, air conditioner, washing machine, rice cooker, gas stove, electric stove, smartphone, microwave, motorbike, car, bike, camera, vacuum cleaner, internet, and laptop/computer). Since the first principal component captured most of the variation of the variables included in the PCA, we then used the first component to derive the wealth index and categorize households into quintiles of different socioeconomic groups based on the wealth index. The first quintile included the poorest households, and the fifth quintile included the wealthiest households. We conducted the PCA separately for urban and rural households since the relative wealth was incomparable between the locations.

We performed a multivariable logistic regression with all variables for smoking cessation (persistent smokers versus quitters) and intention to quit (persistent smokers with the intention to quit versus those without intention at one-year follow-up). All variables included in the regression model for smoking cessation were measured at baseline survey, whereas the regression model of intention to quit included variables measured at follow-up survey. The analysis is presented as adjusted odds ratios (aOR) and 95% confidence interval (95% CI). We further performed a multinomial logistic regression to identify factors associated with the probability of being in the different stages of intention to quit smoking (pre-contemplation, contemplation, and preparation) (reported in risk ratio and 95% CI). Finally, we conducted a sensitivity analysis using the different *p*-value threshold for the regression models (*p *<0.05, *p* < 0.1 and *p* < 0.2). All the analyses were performed in Stata 17 (StataCorp, College Station, TX, USA).

## Results

### Demographic characteristics of persistent smokers and quitters

Of the 1525 participants who participated in both baseline and one-year follow-up, only 15.6% were identified as quitters at the follow-up. Quitters were older than persistent smokers (49.18 vs 45.58 years old, respectively) and had a higher score on the quality of life (VAS scale of 82.05 vs 80.71, respectively). We also observed a higher proportion of quitters among those who self-assessed themselves with good/excellent health, those with a history of chronic disease, and those who did not consume alcohol (Table 1).

**Table 1:** Smoking cessation prevalence by study participants’ characteristics at baseline and follow up^a^

Characteristics	Smoking cessation status at one-year follow-up	
	Persistent smokers	Quitters
	*n* = 1287 (85.2%)	*n* = 238 (14.8%)
**Demographic variable at baseline**		
Region type		
Urban areas	646 (84.9)	122 (15.1)
Rural areas	641 (85.7)	116 (14.3)
Age, mean (SE)	45.58 (13.99)	49.18 (15.20)
Marital status		
Living without partner	146 (87.4)	21 (12.6)
Living with partner	1141 (84.9)	217 (15.1)
Education attainment		
Secondary school completed or lower	554 (84.7)	99 (15.3)
High school completed	429 (85.1)	81 (14.9)
College/University or higher	304 (86.2)	58 (13.8)
Household quintiles based on wealth index		
Quintile I (poorest)	275 (88.1)	41 (11.9)
Quintile II	251 (80.9)	58 (19.1)
Quintile III	253 (84.1)	50 (15.9)
Quintile IV	264 (87.3)	46 (12.7)
Quintile V (richest)	244 (85.8)	43 (14.2)
**Health behaviours at follow-up**		
Self-assessed health status		
Fair	776 (86.5)	124 (13.5)
Worst/Poor	95 (90.1)	12 (9.9)
Good/Excellent	416 (81.9)	102 (18.1)
Quality of life (VAS scale), mean (SE)	80.71 (12.57)	82.05 (13.84)
Ever diagnosis with any chronic disease		
No	1021 (86.7)	167 (13.3)
Yes	266 (79.8)	71 (20.2)
Alcohol consumption		
No	109 (77.9)	30 (22.1)
Yes	1178 (86.0)	208 (14.0)

*SE* Standard error

^a^Sample size for individual characteristics may not be equal to the total due to missing values

Compared to the persistent smokers, quitters smoked fewer cigarettes per day at baseline (11.01 versus 13.90), had a lower HSI (1.66 versus 2.09), and had fewer smoking friends (3.25 versus 3.59). Participants who had more friends or acquaintances who successfully quit smoking or did not have smokers in their family had a higher likelihood of quitting smoking themselves. Moreover, individuals who only smoked cigarettes had a higher likelihood of quitting compared to those who were dual smokers. Additionally, we observed that a higher percentage of quitters held positive attitudes toward smoking compared to those who continued to smoke. We observed similar trends in their belief, opinions about smoking and perceived positive impact of tobacco control measures, as shown in Table 2.

**Table 2:** Smoking cessation prevalence by study participants’ characteristics at baseline and follow-up survey^a^

Characteristics	Smoking cessation at one-year follow-up, n (%)	
	Persistent smokers	Quitters
	*n* = 1287 (85.2%)	*n* = 238 (14.8%)
**Smoking behaviours at baseline**		
Tobacco smoke type		
Cigarette smoking only	1035 (85.0)	213 (15.0)
Dual users	252 (86.4)	25 (13.6)
Smoking duration (years)		
≤ 5 years	117 (81.7)	30 (18.3)
> 5–10 years	153 (91.6)	19 (8.4)
> 10 years	1017 (84.8)	189 (15.2)
Cigarettes smoked per day, mean (SE)	13.90 (8.80)	11.01 (8.63)
Cigarettes smoked per day		
≤ 10 cigarettes	661 (81.8)	144 (18.2)
> 10–20 cigarettes	525 (89.1)	81 (10.9)
> 20–30 cigarettes	57 (85.2)	8 (14.8)
≥ 31 cigarettes	44 (92.8)	4 (7.2)
The heaviness of smoking index, mean (SE)	2.09 (1.62)	1.66 (1.63)
Number of smokers among five closest friends, mean (SE)	3.59 (1.44)	3.25 (1.56)
Number of friends/acquaintances who quit smoking successfully		
No	658 (88.9)	82 (11.1)
One person	173 (85.8)	30 (14.2)
Two or more people	420 (80.5)	110 (19.5)
Smokers in the families		
No	1019 (83.5)	207 (16.5)
Yes	268 (90.0)	31 (10.0)
Tobacco-related knowledge		
Quartile I (lowest)	313 (88.1)	49 (11.9)
Quartile II	367 (88.9)	50 (11.1)
Quartile III	258 (81.3)	59 (18.7)
Quartile IV (highest)	349 (82.2)	80 (17.8)
**Attitude & belief at baseline**		
Self-efficacy to quit smoking		
Not at all	499 (87.5)	68 (12.5)
Somewhat	187 (87.4)	34 (12.6)
A lot	587 (82.8)	132 (17.2)
Perceived health benefits of quitting		
Not at all	336 (89.0)	49 (11.0)
A lot	921 (83.5)	185 (16.5)
Worried about health in the future		
Not at all	270 (91.4)	32 (8.6)
Somewhat	476 (84.8)	86 (15.2)
A lot	529 (82.5)	118 (17.5)
Overall opinion of smoking		
Good	158 (86.6)	23 (13.4)
Bad	1119 (85.0)	215 (15.0)
**Perceived impacts of tobacco control policies assessed at follow-up**		
Smoke-free policies		
No	948 (88.9)	137 (11.1)
Yes	336 (75.7)	100 (24.3)
Cessation support program		
No	857 (86.2)	145 (13.8)
Yes	195 (81.1)	47 (18.9)
Health warning labels		
No	981 (89.4)	122 (10.6)
Yes	304 (73.7)	115 (26.3)
Anti-smoking advertising		
No	1169 (86.1)	199 (13.9)
Yes	105 (75.6)	37 (24.4)
Tobacco taxation		
No	957 (87.6)	146 (12.4)
Yes	315 (81.0)	72 (19.0)

*SE* Standard error

^a^Sample size for individual characteristics may not be equal to the total due to missing values

**Fig. 2:**
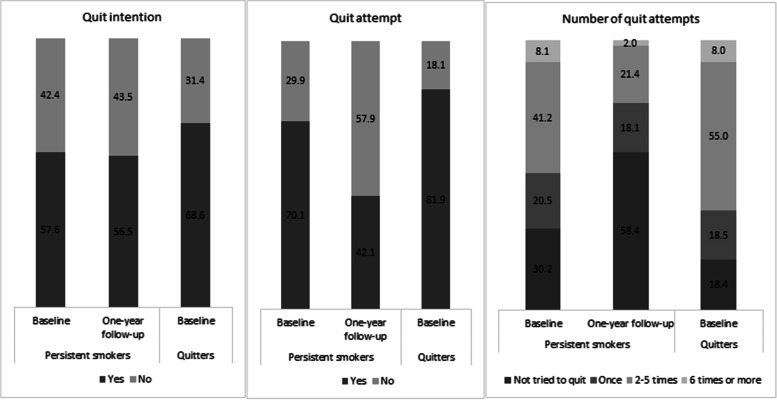
Intention to quit, quit attempt, and the number of quit attempts during a year among persistent smokers assessed at baseline and one-year follow-up (*n* = 1287) and quitters assessed at baseline (*n* = 238)

The percentages of people who reported their intention to quit and quit attempts in the baseline survey were much higher among quitters (68.6% and 81.9%, respectively) than persistent smokers (57.6% and 70.1%, respectively). Among persistent smokers, fewer reported quitting attempts in the follow-up than in the baseline (42.1% versus 70.1%). They also made a smaller number of quit attempts (Fig. 2).

### Factors associated with smoking cessation

As shown in Table 3, as the number of cigarettes smoked per day increased, the perceived chance of successfully quitting declined (aOR = 0.97, 95% CI: 0.94, 0.99). Further, quitters were those who had several attempts to quit smoking previously (aOR = 2.10; 95% CI: 1.15, 3.82). Though not statistically significance in all sub-groups, smoking cessation was shown to increase with the tobacco-related knowledge and among those had intention to quit smoking. Also, none of the perceived impact of tobacco control policy variables were statistically significant association with the smoking cessation.

**Table 3:** Individual-, social- and policy- factors associated with smoking cessation after one-year follow-up (*n* = 1423)

	**Smoking cessation**
	**aOR (95% CI)**
Region type	
Urban areas	REF
Rural areas	1.04 (0.61—1.79)
Age group	
18–39	REF
≥ 40	1.02 (0.69—1.49)
Marital status	
Living without partner	REF
Living with partner	1.15 (0.60—2.22)
Education attainment	
Secondary school completed or lower	REF
High school completed	0.99 (0.60—1.66)
College/University or higher	0.97 (0.52—1.80)
Household wealth index	
Quintile I (poorest)	0.88 (0.48—1.60)
Quintile II	1.55 (0.78—3.06)
Quintile III	1.32 (0.71—2.45)
Quintile IV	0.80 (0.37—1.72)
Quintile V (richest)	REF
Tobacco smoke type	
Cigarette smoking only	1.48 (0.84—2.62)
Dual use	REF
Smoking duration	
≤ 5 years	1.20 (0.59—2.44)
> 5–10 years	0.46 (0.20—1.02)
> 10 years	REF
Cigarette smoked per day	**0.97 (0.94—0.99)**
Self-assessed health status	
Fair	REF
Worst/Poor	1.02 (0.52—1.99)
Good/Excellent	0.88 (0.55—1.41)
Number of smokers among 5 closest friends	0.95 (0.85—1.07)
Tobacco-related knowledge	
Quartile I (lowest)	REF
Quartile II	0.70 (0.36—1.39)
Quartile III	1.50 (0.75—3.00)
Quartile IV (highest)	1.40 (0.74—2.65)
Self-efficacy to quit smoking	
Not at all	REF
Somewhat	0.89 (0.47—1.69)
A lot	1.16 (0.68—2.00)
Health benefits of quitting	
Not at all	REF
A lot	1.43 (0.79—2.80)
Worried about future health	
Not at all	REF
Somewhat	1.61 (0.85—3.02)
A lot	1.49 (0.84—2.64)
Opinion of smoking	
Good	REF
Bad	0.75 (0.35—1.60)
Intention to quit	
No	REF
Yes	1.14 (0.67—1.93)
Number of quit attempts during the previous year	
Not tried to quit	REF
Once	1.44 (0.74—2.81)
2–5 times	**2.10 (1.15—3.82)**
6 times or more	1.10 (0.45—2.67)
Health warning labels	
No	REF
Yes	0.94 (0.62—1.44)
Anti-smoking advertising	
No	REF
Yes	0.73 (0.29—1.81)

*aOR* Adjusted odds ratio, *95% CI* 95% confidence interval, *REF* Reference group

Adjusted for all variables in the final model

Bold values denote statistical significance at the *p* < .05 level

### Factors associated with the intention to quit

We observed that persistent smokers who perceived that tobacco control policies, including health warning labels (aOR = 2.65; 95% CI: 1.26, 5.58) and tobacco taxation (aOR = 2.39; 95% CI: 1.26, 4.55) had a positive impact on their smoking habit also expressed more intentions to quit. Further, those who diagnosed with chronic disease (aOR = 2.18, 95% CI: 1.20, 3.96) were positively associated with the intention to quit smoking. Also, this association was stronger with increased number of quit attempts, higher tobacco-related knowledge, greater self-efficacy to quit, or more worries about their future health (Table 4).

**Table 4:** Individual-, social- and policy- factors associated with the intention to quit smoking among persistent smokers (*n* = 957)

	**Intention to quit**
	**aOR (95% CI)**
Region type	
Urban areas	REF
Rural areas	**2.59 (1.73—3.88)**
Age group	
18–39	REF
≥ 40	0.87 (0.51—1.49)
Marital status	
Living without partner	REF
Living with partner	0.86 (0.41—1.79)
Education attainment	
Secondary school completed or lower	REF
High school completed	1.32 (0.65—2.67)
College/University or higher	1.00 (0.54—1.86)
Household wealth index	
Quintile I (poorest)	1.22 (0.60—2.48)
Quintile II	1.56 (0.74—3.29)
Quintile III	1.40 (0.60—3.25)
Quintile IV	0.94 (0.37—2.37)
Quintile V (richest)	REF
Tobacco smoke type	
Cigarette smoking only	1.00 (0.69—1.45)
Dual use	REF
Smoking duration	
≤ 5 years	2.98 (0.92—9.68)
> 5–10 years	1.43 (0.74—2.76)
> 10 years	REF
Cigarette smoked per day	1.00 (0.97—1.02)
Self-assessed health status	
Fair	REF
Worst/Poor	**0.32 (0.12—0.88)**
Good/Excellent	0.75 (0.43—1.31)
Quality of life (VAS scale)	0.99 (0.97 – 1.00)
Ever been diagnosed with any chronic disease	
No	REF
Yes	**2.18 (1.20—3.96)**
Alcohol consumption	
No	REF
Yes	1.64 (0.86—3.12)
Number of smokers among 5 closest friends	0.98 (0.82—1.16)
The number of friends/acquaintances who quit smoking successfully	
No	REF
One person	0.92 (0.50—1.70)
2 or more people	1.24 (0.81—1.90)
Smokers in the families	
No	1.32 (0.75—2.33)
Yes	
Number of quit attempts during the previous year	
Not tried to quit	REF
Once	**3.60 (1.74—7.46)**
2–5 times	**6.24 (3.24—12.04)**
6 times or more	**5.45 (1.41—21.09)**
Tobacco-related knowledge	
Quartile I (lowest)	REF
Quartile II	1.70 (0.95—3.16)
Quartile III	**2.16 (1.04—4.46)**
Quartile IV (highest)	**2.27 (1.04—4.93)**
Self-efficacy to quit smoking	
Not at all	REF
Somewhat	**3.73 (2.05—6.78)**
A lot	**3.82 (2.14—6.80)**
Health benefits of quitting	
Not at all	REF
A lot	1.23 (0.70—2.18)
Worried about future health	
Not at all	REF
Somewhat	**2.08 (1.25—3.45)**
A lot	**3.04 (1.66—5.56)**
Opinion of smoking	
Good	REF
Bad	1.53 (0.76—3.09)
Smoke-free policies	
No	REF
Yes	1.23 (0.65—2.34)
Cessation support program	
No	REF
Yes	1.04 (0.58—1.88)
Health warning labels	
No	REF
Yes	**2.65 (1.26—5.58)**
Anti-smoking advertising	
No	REF
Yes	1.86 (0.69—4.99)
Tobacco taxation	
No	REF
Yes	**2.39 (1.26—4.55)**

aOR, Adjusted odds ratio; 95% CI, 95% confidence interval; REF, reference value

Adjusted for all variables in the final model

Bold values denote statistical significance at the *p* < .05 level

### Sensitivity analyses

Our sensitivity analysis to investigate the robustness of the findings comparing the regression models that statistically significant at different *p*-values (Supplementary Tables 2 and 3). The multinomial logistic regression analysis revealed some associated factors across the three models of different stages of intention to quit (Supplementary Table 4). Overall, intention to quit at any stage was associated with being diagnosed with chronic disease, had greater self-efficacy, more worried about their future health, had more quit attempts. However, some factors were associated in one model only, including between had more tobacco-related knowledge or smoke-free policy impacts and intention to quit within this month, and between those who had the intention to quit within the next six months or in the future and health warning label and tobacco taxation policies.

## Discussion

In this study, we examined the individual-, social- and policy-level factors associated with quitting behaviours of adult Vietnamese smokers. Only one-sixth of the smokers had quit successfully for at least 30 consecutive days at one-year follow-up. Factors associated with smoking cessation included having fewer cigarettes smoked daily and several attempts to quit smoking. Among the persistent smokers, over half of them who did not quit at one year of follow-up intended to quit smoking. Factors associated with intention to quit included self-reported diagnosis of chronic diseases, having previous quitting attempts, having more tobacco-related knowledge, having greater self-efficacy, worrying about their future health, and perceived positive influence by the health warning labels and tobacco taxation policies.

Our findings that more than half of adult smokers intended to quit smoking is comparable to the results in the national GATS survey in Vietnam [1], but much higher than the reported number in other Asian countries [7, 9, 21, 22]. Still, it is lower than the intention to quit reported in high-income countries (ranging from 67.0% in the UK to more than 80.0% in Canada) [23]. Different definitions employed in different studies could explain the differences in the proportions of intention to quit observed. Furthermore, our study participants had higher consumption levels (CPD of 13.7) and higher nicotine dependence levels than those in another study [7]. This could explain the discrepancies between our findings and the other studies since smokers with high levels of nicotine dependence had less intention to quit [23].

Consistent with studies in other countries [7–9, 24], we also observed that quit attempts are positively associated with the intention to quit and smoking cessation. We found that persistent smokers who intended to quit smoking also expressed greater self-efficacy, a positive attitude towards their future health, and an unfavourable opinion of smoking, in line with earlier findings [8–10, 24, 25]. We also observed that smoking cessation prevalence was higher in those with good/excellent health or a chronic disease diagnosis.

Our longitudinal data indicated a slight decrease in the proportion of persistent smokers who attempted quitting and decreased self-efficacy between the baseline and the follow-up. A plausible explanation for these findings is that it takes many smokers several quit attempts before a successful smoking cessation. Earlier prospective study also confirmed that smokers’ self-efficacy was necessary and sufficient for their smoking cessation [26]. Further, our multinomial regression results again confirmed the association between the number of quit attempts or self-efficacy across different stages of smoking cessation. The associations were found to be stronger if a smoker was more ready for the cessation process (preparation stage vs contemplation or precontemplation stage). Some might try quitting less often after multiple failures; thus, it is essential to keep the motivation to quit even after failure(s) [19]. These findings imply that fostering smokers’ willpower and self-efficacy, especially those contemplating quitting and those with a history of quitting attempts, may be necessary.

Further, our one-year smoking cessation percentage of around 15% was in line with the report in India (14.2%) [27] and Indonesia (12.3%) [28], but slightly lower than that reported in high-income nations like the USA (over 25.0%) [29], and Poland (30.4–37.9%) [30]. The difference might be driven by differences in (1) assessment of the smoking behaviours (smoking duration, nicotine dependence, stage of addiction), (2) the availability of cessation support programs, and (3) the level of tobacco control policies implemented in each country. These differences might also reflect cultural differences and social norms since male smoking has long been accepted by society in Vietnam and plays an integral role in the male social culture [31]. Future research could dig deeper into the role of families’, peers’ and society’s norms on male smokers’ behaviour change, which is in line with a recent suggestion that taking the role of culture and social norms should be strictly considered when formulating public health policies [32].

Besides, smoking duration and frequency, which seem to drive the smoking cessation phase, are well-documented in the literature [7–9, 14]. Our findings agreed with these findings as the increase in the number of cigarettes smoked daily was associated with a decrease in smoking cessation. Then, the results suggest that smoking intake level possibly influences quit behaviour. However, low-level smokers may not perceive themselves as smokers or addicted to nicotine. Consequently, light smokers may not recognize the association between adverse health outcomes and low-level smoking [33]. Nicotine in tobacco products is a well-known addictive chemical; thus, even occasional or light smoking can lead to heavier consumption in the longer term, making smoking cessation challenging. Therefore, cessation support programs could also actively target those still in an early stage of addiction to support them to quit smoking earlier.

Different from previous studies in Sri Lanka [34], Hong Kong [35], and Saudi Arabia [36], we did not find any significant social factors such as friends, family members, or acquaintances on persistent smokers’ intention to quit and smoking cessation. Participants’ age could partly explain this difference since the earlier studies surveyed younger participants, who were more likely influenced by their peers than the older [35, 36]. Future qualitative research could explore what motivate smokers to quit or have intention to quit smoking and the role of friends/family in their smoking cessation behaviour.

During the mid-1990s, Vietnam put more strenuous efforts into more robust tobacco activities by enacting various tobacco control policies. The national commitment culminated with the highly ambitious National Tobacco Control Policy in 2000 [11–13]. All five tobacco control policies in this study showed a positive association with quitting behaviours, though only two policies showed statistically significant results. First, smoking cessation behaviour has started from not wanting to think or see about the health warning labels, changing smokers’ behaviours like forgoing cigarettes and avoiding seeing a cigarette pack finally quitting smoking as mentioned in earlier studies [37, 38]. In systematic reviews that include both observation and longitudinal studies, the effectiveness of the warning labels in triggering a positive change in smokers’ attitudes and behaviours have been reported [39, 40]. However, the effects of these warnings varied by different demographic groups, and its effectiveness was sustained only in the first two years and then decreased over time [41]. Therefore, replacing the 8-year-old graphic labels aimed to discourage people from continuing to smoke could be an effective public health action. Upcoming studies could also examine the effects of the health warning labels on different demographic groups in the Vietnamese population to further understand its impacts and have more ideas on the specific target groups for smoking cessation intervention in Vietnam. Second, smokers’ intention to quit increased with the positively perceived impact of tobacco taxation. However, the cigarette price in Vietnam has even become more affordable relative to income since 2010 [4], which impedes reaching the national target of reducing smoking prevalence to a maximum of 39%. Though mandated to protect population health through implementing policies to reduce tobacco use, the Vietnam government has a conflict of interest since tobacco companies are state-owned and dominates the national cigarette market with a 60% market share. This conflict of interest between receiving benefits from tobacco product manufacturing and being in charge of controlling tobacco consumption may pose challenges and obstacles for the government to plan and execute effective tobacco control programmes in Vietnam. One means for addressing this issue could be to establish a code of conduct or stricter tobacco legislation from tobacco industry interference.

On the other hand, we did not find any significant association between other tobacco control policies, such as cessation support programs or smoke-free policy with smoking cessation behaviours. It could be partly expected since cessation services have existed only in some healthcare facilities throughout Vietnam. In addition, the total tax imposed on cigarettes amounts to only 36% of the retail price. This figure falls significantly below the WHO's recommended threshold of 75% [4, 13]. Even not statistically significant, we found a positive relationship between the perception of smoke-free policies and intentions to quit, consistent with various surveys in Vietnam and worldwide [42, 43]. A previous survey conducted in Vietnam underscored that these smoke-free regulations reminded and encouraged smokers to stop smoking [42]. Besides, the anti-smoking regulation is perceived to discourage smoking initiation among youths and spare people from second-hand smoke [43]. Therefore, the Vietnamese government has shown a solid commitment to expanding the smoke-free policies to other places, including restaurants and hotels, to maximize the impact.

### Strengths and limitations

The current study is the first longitudinal study to assess smoking cessation behaviour and the impacts of tobacco control policies on quitting behaviours among adult male smokers in Vietnam. We employed a standardized questionnaire from the ITC, which was well-established to evaluate tobacco control policies globally [18]. This prospective design allowed us to follow smokers’ smoking behaviour and their attitude/self-efficacy change over time that other smoking cessation cross-sectional studies in Vietnam could not. Further, the sampling weights were calculated and utilized to reduce or minimize any possible bias.

However, our findings should not be generalized as we only sampled from two areas in Hanoi. Further, our sample is limited to only male smokers in two purposively selected districts of Hanoi, and hence, we could not draw inferences about female smokers or male smokers overall. Further, following the participants for one year only, we cannot capture the dynamics of quitting behaviours at multiple times. Those who quit during the last 30 days might not maintain their longer-term abstinence, and others might have quit for over 30 days in the year. All information, including smoking cessation, was self-reported and not validated with an objective measurement. We also evaluated the perceived impact of tobacco control policies among smokers only, and hence we cannot generalize these findings to the general population. Our study measured perceived impact only, which could be affected by a social desirability bias and couldn’t reflect the actual impact of these policies on smokers’ behaviour. Finally, this quantitative survey did not study smoking-related social norms and social context. Combining qualitative and quantitative studies in a mixed-method design will strengthen future tobacco cessation studies.

Our findings carry several implications. Given that quitting behaviour is associated with some individual-, social- and policy factors, these factors should be considered in designing an effective smoking cessation program. Future research using a mixed-methods approach could yield richer information on these aspects to design relevant public health interventions that promote reductions in initiation and smoking cessation among smokers. Further, smokers appear to perceive the impact from tobacco control policies, such as smoke-free regulations and warning labels on cigarette packages, in a way of encouraging them to quit smoking. Therefore, reinforcing these policies may encourage people to quit smoking and curb the tobacco pandemic burden. The impact of these policies may differ between different groups and/or contexts, which could be explored in future research.

## Conclusion

This study identified multi-dimensional factors to tobacco cessation among adult male smokers in Vietnam. Interventions aimed at increasing smoking cessation should focus on individual, social and policy factors. Our results indicate that persistent smokers who are contemplating quitting, may benefit from having better knowledge of tobacco harms or greater self-efficacy. Health warning labels and tobacco taxation should be maintained and promoted as they are perceived as particularly useful for persistent smokers’ intention to quit.

### Supplementary Information

**Additional file 1: Supplement 1. **Variables definition.

**Additional file 2: Suppl 2.** Multivariable logistic regression analysis for smoking cessation (sensitivity analysis using the different *p*-values).

**Additional file 3: Suppl 3.** Multivariable logistic regression analysis for intention to quit (sensitivity analysis using using the different *p*-values).

**Additional file 4: Suppl 4.** Multinomial logistic regression analysis for intention to quit.

## Data Availability

In each country participating in the International Tobacco Control Policy Evaluation (ITC) Project, the data are jointly owned by the lead researcher(s) in that country and the ITC Project at the University of Waterloo. Data from the ITC Project are available to approved researchers two years after the date of issuance of cleaned data sets by the ITC Data Management Centre. Researchers interested in using ITC data are required to apply for approval by submitting an International Tobacco Control Data Repository (ITCDR) request application and subsequently to sign an ITCDR Data Usage Agreement. The criteria for data usage approval and the contents of the Data Usage Agreement are described online (http://www.itcproject.org).

## Abbreviations

GATS: Global Adult Tobacco Survey
WHO: World Health Organization
The ITC Project: The International Tobacco Control Policy Evaluation Project
PSU: Primary sampling units
VAS: Visual analogue scale
CPD: Cigarettes smoked per day
HIS: Heaviness smoking index
SE: Standard error
PCA: Principal component analysis
aOR: Adjusted odds ratios
95% CI: 95% Confidence interval
REF: Reference
