# Supplementary material for: Individual-, social- and policy- factors associated with smoking cessation among adult male cigarette smokers in Hanoi, Vietnam: a longitudinal study
Source: BMC Public Health. 2023 Sep 28;23:1883. doi: 10.1186/s12889-023-16781-7 (PMC10540420; doi:10.1186/s12889-023-16781-7)
Supplement: Supplementary file 1 — Additional file 1: Supplement 1. Variables definition. [file 12889_2023_16781_MOESM1_ESM.docx]

**Supplement 1: Variables definition**

| **Construct** | **Questions** | **Categories in the analysis (original categories)** |
| --- | --- | --- |
| **Attitudes and beliefs of smoking/quitting** | | |
| Self-efficacy to quit smoking | If you decided to give up smoking completely in the next six months, how sure are you that you would succeed? | 0. Not at all (not at all sure)  1. Somewhat (somewhat sure)  2. A lot (very sure, extremely sure) |
| Health benefits of quitting | How much do you think you would benefit from health and other gains if you were to quit smoking permanently in the next six months? | 0. Not at all (not at all, a little)  1. A lot (very much) |
| Worres about future health | How worried are you, if at all, that smoking will damage your health in the future? | 0. Not at all (not worried at all)  1. Somewhat (somewhat worried)  2. A lot (very worried) |
| Opinion of smoking | What is your overall opinion of smoking? | 0. Good/Neutral (good, very good, neutral)  1. Bad (bad, very bad) |
| **Perceived impact of tobacco control policies** | | |
| Smoke-free policies | To what extent do smoke-free policies in public places make you more likely to QUIT smoking? | 0. No (not at all, a little)  1. Yes (a lot) |
| Cessation support | To what extent do the cessation support or quit-smoking medications make you more likely to quit smoking? | 0. No (not at all, a little)  1. Yes (a lot) |
| Health warning labels | To what extent do the health warnings on cigarette packs make you more likely to quit smoking? | 0. No (not at all, a little)  1. Yes (a lot) |
| Anti-smoking advertising | Overall, what do these anti-smoking campaigns make you think about quitting smoking? | 0. No (not at all, less likely)  1. Yes (More likely) |
| Tobacco taxation | To what extent do the increase in tobacco taxation make you more likely to quit smoking? | 0. No (not at all, a little)  1. Yes (a lot) |

Note: All “do not know” responses were coded as missing.
