# Supplementary material for: Individual-, social- and policy- factors associated with smoking cessation among adult male cigarette smokers in Hanoi, Vietnam: a longitudinal study
Source: BMC Public Health. 2023 Sep 28;23:1883. doi: 10.1186/s12889-023-16781-7 (PMC10540420; doi:10.1186/s12889-023-16781-7)
Supplement: Supplementary file 2 — Additional file 2: Suppl 2. Multivariable logistic regression analysis for smoking cessation (sensitivity analysis using the different p-values). [file 12889_2023_16781_MOESM2_ESM.docx]

**Suppl 2: Multivariable logistic regression analysis for smoking cessation (sensitivity analysis using the different *p*-value)**

|  | **Smoking cessation (n=1423)** |
| --- | --- |
|  | **aOR (95% CI)** |
| Region type |  |
| Urban areas | REF |
| Rural areas | 1.04 (0.61 - 1.79) |
| Age group |  |
| 18-39 | REF |
| ≥40 | 1.02 (0.69 - 1.49) |
| Marital status |  |
| Living without partner | REF |
| Living with partner | 1.15 (0.60 - 2.22) |
| Education attainment |  |
| Secondary school completed or lower | REF |
| High school completed | 0.99 (0.60 - 1.66) |
| College/University or higher | 0.97 (0.52 - 1.80) |
| Household wealth index |  |
| Quintile I (poorest) | 0.88 (0.48 - 1.60) |
| Quintile II | 1.55 (0.78 - 3.06) |
| Quintile III | 1.32 (0.71 - 2.45) |
| Quintile IV | 0.80 (0.37 - 1.72) |
| Quintile V (richest) | REF |
| Tobacco smoke type |  |
| Cigarette smoking only | 1.48* (0.84 - 2.62) |
| Dual use | REF |
| Smoking duration |  |
| ≤5 years | 1.20 (0.59 - 2.44) |
| >5-10 years | 0.46** (0.20 - 1.02) |
| >10 years | REF |
| Cigarette smoked per day | **0.97*** (0.94 - 0.99)** |
| Self-assessed health status |  |
| Fair | REF |
| Worst/Poor | 1.02 (0.52 - 1.99) |
| Good/Excellent | 0.88 (0.55 - 1.41) |
| Number of smokers among 5 closest friends | 0.95 (0.85 - 1.07) |
| Tobacco-related knowledge |  |
| Quartile I (lowest) | REF |
| Quartile II | 0.70 (0.36 - 1.39) |
| Quartile III | 1.50 (0.75 - 3.00) |
| Quartile IV (highest) | 1.40 (0.74 - 2.65) |
| Self-efficacy to quit smoking |  |
| Not at all | REF |
| Somewhat | 0.89 (0.47 - 1.69) |
| A lot | 1.16 (0.68 - 2.00) |
| Health benefits of quitting |  |
| Not at all | REF |
| A lot | 1.43 (0.79 - 2.80) |
| Worried about future health |  |
| Not at all | REF |
| Somewhat | 1.61* (0.85 - 3.02) |
| A lot | 1.49* (0.84 - 2.64) |
| Opinion of smoking |  |
| Good | REF |
| Bad | 0.75 (0.35 - 1.60) |
| Intention to quit |  |
| No | REF |
| Yes | 1.14 (0.67 - 1.93) |
| Number of quit attempts during the previous year |  |
| Not tried to quit | REF |
| Once | 1.44 (0.74 - 2.81) |
| 2-5 times | **2.10*** (1.15 - 3.82)** |
| 6 times or more | 1.10 (0.45 - 2.67) |
| Health warning labels |  |
| No | REF |
| Yes | 0.94 (0.62 - 1.44) |
| Anti-smoking advertising |  |
| No | REF |
| Yes | 0.73 (0.29 - 1.81) |

Significant at * p<0.2, ** p<0.1, *** p<0.05
