# Supplementary material for: Individual-, social- and policy- factors associated with smoking cessation among adult male cigarette smokers in Hanoi, Vietnam: a longitudinal study
Source: BMC Public Health. 2023 Sep 28;23:1883. doi: 10.1186/s12889-023-16781-7 (PMC10540420; doi:10.1186/s12889-023-16781-7)
Supplement: Supplementary file 3 — Additional file 3: Suppl 3. Multivariable logistic regression analysis for intention to quit (sensitivity analysis using using the different p-values). [file 12889_2023_16781_MOESM3_ESM.docx]

**Suppl 3: Multivariable logistic regression analysis for intention to quit** **(sensitivity analysis using using the different *p*-values)**

|  | **Full model (n=928)** |
| --- | --- |
|  | **aOR (95% CI)** |
| Region type |  |
| Urban areas | REF |
| Rural areas | **2.59*** (1.73 - 3.88)** |
| Age group |  |
| 18-39 | REF |
| ≥40 | 0.87 (0.51 - 1.49) |
| Marital status |  |
| Living without partner | REF |
| Living with partner | 0.86 (0.41 - 1.79) |
| Education attainment |  |
| Secondary school completed or lower | REF |
| High school completed | 1.32 (0.65 - 2.67) |
| College/University or higher | 1.00 (0.54 - 1.86) |
| Household wealth index |  |
| Quintile I (poorest) | 1.22 (0.60 - 2.48) |
| Quintile II | 1.56 (0.74 - 3.29) |
| Quintile III | 1.40 (0.60 - 3.25) |
| Quintile IV | 0.94 (0.37 - 2.37) |
| Quintile V (richest) | REF |
| Tobacco smoke type |  |
| Cigarette smoking only | 1.00 (0.69 - 1.45) |
| Dual use | REF |
| Smoking duration |  |
| ≤5 years | 2.98** (0.92 - 9.68) |
| >5-10 years | 1.43 (0.74 - 2.76) |
| >10 years | REF |
| Cigarette smoked per day | 1.00 (0.97 - 1.02) |
| Self-assessed health status |  |
| Fair | REF |
| Worst/Poor | **0.32*** (0.12 - 0.88)** |
| Good/Excellent | 0.75 (0.43 - 1.31) |
| Quality of life (VAS scale) | 0.99* (0.97 – 1.00) |
| Ever been diagnosed with any chronic disease |  |
| No | REF |
| Yes | **2.18*** (1.20 - 3.96)** |
| Alcohol consumption |  |
| No | REF |
| Yes | 1.64* (0.86 - 3.12) |
| Number of smokers among 5 closest friends | 0.98 (0.82 - 1.16) |
| The number of friends/acquaintances who quit smoking successfully. |  |
| No | REF |
| One person | 0.92 (0.50 - 1.70) |
| 2 or more people | 1.24 (0.81 - 1.90) |
| Smokers in the families |  |
| No | 1.32 (0.75 - 2.33) |
| Yes |  |
| Number of quit attempts during the previous year |  |
| Not tried to quit | REF |
| Once | **3.60*** (1.74 - 7.46)** |
| 2-5 times | **6.24*** (3.24 - 12.04)** |
| 6 times or more | **5.45*** (1.41 - 21.09)** |
| Tobacco-related knowledge |  |
| Quartile I (lowest) | REF |
| Quartile II | 1.70** (0.95 - 3.16) |
| Quartile III | **2.16*** (1.04 - 4.46)** |
| Quartile IV (highest) | **2.27*** (1.04 - 4.93)** |
| Self-efficacy to quit smoking |  |
| Not at all | REF |
| Somewhat | **3.73*** (2.05 - 6.78)** |
| A lot | **3.82*** (2.14 - 6.80)** |
| Health benefits of quitting |  |
| Not at all | REF |
| A lot | 1.23 (0.70 - 2.18) |
| Worried about future health |  |
| Not at all | REF |
| Somewhat | **2.08*** (1.25 - 3.45)** |
| A lot | **3.04*** (1.66 - 5.56)** |
| Opinion of smoking |  |
| Good | REF |
| Bad | 1.53 (0.76 - 3.09) |
| Smoke-free policies |  |
| No | REF |
| Yes | 1.23 (0.65 - 2.34) |
| Cessation support program |  |
| No | REF |
| Yes | 1.04 (0.58 - 1.88) |
| Health warning labels |  |
| No | REF |
| Yes | **2.65*** (1.26 - 5.58)** |
| Anti-smoking advertising |  |
| No | REF |
| Yes | 1.86 (0.69 - 4.99) |
| Tobacco taxation |  |
| No | REF |
| Yes | **2.39*** (1.26 - 4.55)** |

Significant at * p<0.2, ** p<0.1, *** p<0.05
