# Supplementary material for: Individual-, social- and policy- factors associated with smoking cessation among adult male cigarette smokers in Hanoi, Vietnam: a longitudinal study
Source: BMC Public Health. 2023 Sep 28;23:1883. doi: 10.1186/s12889-023-16781-7 (PMC10540420; doi:10.1186/s12889-023-16781-7)
Supplement: Supplementary file 4 — Additional file 4: Suppl 4. Multinomial logistic regression analysis for intention to quit. [file 12889_2023_16781_MOESM4_ESM.docx]

**Suppl 4: Multinomial logistic regression analysis for intention to quit**

|  | **Within this month** | **Within the next 6 months** | **In the future** |
| --- | --- | --- | --- |
| Region type |  |  |  |
| Urban areas | REF | REF | REF |
| Rural areas | **3.55 (2.07 - 6.06)** | 1.21 (0.59 - 2.49) | **3.09 (2.00 - 4.79)** |
| Age group |  |  |  |
| 18-39 | REF | REF | REF |
| ≥40 | 0.99 (0.45 - 2.21) | 0.25 (0.10 - 0.62) | 1.32 (0.79 - 2.19) |
| Marital status |  |  |  |
| Living without partner | REF | REF | REF |
| Living with partner | 0.73 (0.30 - 1.81) | 1.36 (0.48 - 3.87) | 0.74 (0.33 - 1.66) |
| Education attainment |  |  |  |
| Secondary school completed or lower | REF | REF | REF |
| High school completed | 1.28 (0.58 - 2.84) | 1.49 (0.62 - 3.61) | 1.32 (0.64 - 2.75) |
| College/University or higher | 0.89 (0.39 - 2.03) | 0.73 (0.34 - 1.58) | 1.08 (0.56 - 2.08) |
| Household wealth index |  |  |  |
| Quintile I (poorest) | 1.92 (0.57 - 6.50) | 1.18 (0.31 - 4.56) | 1.07 (0.55 - 2.08) |
| Quintile II | 1.96 (0.61 - 6.26) | 2.55 (0.68 - 9.58) | 1.26 (0.67 - 2.37) |
| Quintile III | 2.58 (0.89 - 7.51) | 1.52 (0.44 - 5.27) | 1.25 (0.57 - 2.74) |
| Quintile IV | 1.00 (0.23 - 4.38) | 0.53 (0.16 - 1.73) | 1.05 (0.44 - 2.51) |
| Quintile V (richest) | REF | REF | REF |
| Tobacco smoke type |  |  |  |
| Cigarette smoking only | 1.16 (0.62 - 2.20) | 1.54 (0.82 - 2.89) | 0.96 (0.63 - 1.45) |
| Dual use | REF | REF | REF |
| Smoking duration |  |  |  |
| ≤5 years | 2.44 (0.73 - 8.16) | 0.69 (0.12 - 3.84) | **4.17 (1.19 - 14.6)** |
| >5-10 years | 1.60 (0.67 - 3.82) | 1.49 (0.62 - 3.59) | 1.52 (0.77 - 3.01) |
| >10 years | REF | REF | REF |
| Cigarette smoked per day | 0.95 (0.90 – 1.00) | 1.00 (0.97 - 1.03) | 1.00 (0.98 - 1.02) |
| Self-assessed health status |  |  |  |
| Fair | REF | REF | REF |
| Worst/Poor | 0.31 (0.09 - 1.09) | **0.21 (0.06 - 0.75)** | 0.36 (0.13 - 1.01) |
| Good/Excellent | 0.51 (0.24 - 1.10) | 0.61 (0.29 - 1.30) | 0.87 (0.50 - 1.51) |
| Quality of life (VAS scale) | 0.99 (0.95 - 1.02) | **0.97 (0.95 - 0.99)** | 0.99 (0.98 - 1.01) |
| Ever been diagnosed with any chronic disease |  |  |  |
| No | REF | REF | REF |
| Yes | **2.88 (1.13 - 7.34)** | **3.19 (1.26 - 8.06)** | **1.75 (1.04 - 2.96)** |
| Alcohol consumption |  |  |  |
| No | REF | REF | REF |
| Yes | 1.11 (0.34 - 3.63) | 1.30 (0.57 - 2.96) | **2.21 (1.12 - 4.36)** |
| Number of smokers among 5 closest friends | 0.95 (0.73 - 1.23) | 0.83 (0.66 - 1.04) | 1.04 (0.87 - 1.25) |
| The number of friends/acquaintances who quit smoking successfully. |  |  |  |
| No | REF | REF | REF |
| One person | 0.59 (0.18 - 1.93) | 0.96 (0.32 - 2.87) | 0.95 (0.54 - 1.68) |
| 2 or more people | 1.63 (0.83 - 3.20) | 1.26 (0.69 - 2.31) | 1.20 (0.73 - 1.98) |
| Smokers in the families |  |  |  |
| No | 0.99 (0.42 - 2.35) | 1.55 (0.74 - 3.25) | 1.41 (0.84 - 2.36) |
| Yes | REF | REF | REF |
| Number of quit attempts during the previous year |  |  |  |
| Not tried to quit | REF | REF | REF |
| Once | **4.42 (2.05 - 9.54)** | **4.50 (1.77 - 11.4)** | **3.35 (1.52 - 7.39)** |
| 2-5 times | **11.9 (4.99 - 28.3)** | **6.86 (2.72 - 17.2)** | **5.22 (2.73 - 9.97)** |
| 6 times or more | **15.1 (3.26 - 70.6)** | 2.90 (0.20 - 42.7) | 3.37 (0.78 - 14.4) |
| Tobacco-related knowledge |  |  |  |
| Quartile I (lowest) | REF | REF | REF |
| Quartile II | **3.43 (1.29 - 9.11)** | 2.05 (0.66 - 6.39) | 1.40 (0.78 - 2.51) |
| Quartile III | **3.75 (1.47 - 9.55)** | 2.22 (0.73 - 6.71) | 1.99 (0.95 - 4.18) |
| Quartile IV (highest) | **4.47 (1.63 - 12.2)** | 2.81 (0.79 - 9.96) | 1.78 (0.90 - 3.52) |
| Self-efficacy to quit smoking |  |  |  |
| Not at all | REF | REF | REF |
| Somewhat | **20.9 (6.94 - 63.1)** | **3.53 (1.57 - 7.94)** | **2.99 (1.57 - 5.70)** |
| A lot | **23.2 (7.65 - 70.4)** | **5.86 (2.79 - 12.3)** | **2.45 (1.37 - 4.36)** |
| Health benefits of quitting |  |  |  |
| Not at all | REF | REF | REF |
| A lot | 0.97 (0.41 - 2.31) | 1.44 (0.65 - 3.16) | 1.16 (0.67 - 2.01) |
| Worried about future health |  |  |  |
| Not at all | REF | REF | REF |
| Somewhat | **3.92 (1.28 - 11.9)** | **2.87 (1.21 - 6.8)** | 1.75 (0.88 - 3.49) |
| A lot | **3.73 (1.62 - 8.57)** | **2.75 (1.05 - 7.2)** | **3.19 (1.50 - 6.74)** |
| Opinion of smoking |  |  |  |
| Good | REF | REF | REF |
| Bad | 1.68 (0.26 - 10.8) | 1.44 (0.55 - 3.74) | 1.65 (0.81 - 3.37) |
| Smoke-free policies |  |  |  |
| No | REF | REF | REF |
| Yes | **2.87 (1.26 - 6.54)** | 1.35 (0.51 - 3.57) | 1.03 (0.48 - 2.20) |
| Cessation support program |  |  |  |
| No | REF | REF | REF |
| Yes | 1.37 (0.57 - 3.33) | 1.49 (0.55 - 4.02) | 0.87 (0.47 - 1.63) |
| Health warning labels |  |  |  |
| No | REF | REF | REF |
| Yes | 1.74 (0.90 - 3.35) | **2.55 (1.13 - 5.78)** | **2.91 (1.24 - 6.82)** |
| Anti-smoking advertising |  |  |  |
| No | REF | REF | REF |
| Yes | 1.91 (0.54 - 6.70) | 1.94 (0.58 - 6.54) | 1.75 (0.65 - 4.69) |
| Tobacco taxation |  |  |  |
| No | REF | REF | REF |
| Yes | 2.52 (0.85 - 7.46) | **2.43 (1.03 - 5.77)** | **2.44 (1.27 - 4.66)** |

Bold values denote statistical significance at p<0.05,
